# A Case of Gastrointestinal Mucormycosis in a Critically Ill Patient

**DOI:** 10.7759/cureus.108747

**Published:** 2026-05-12

**Authors:** Aldon Whitehead, Ruchira Khosavanna, Tyler Luu, Maricar Malinis

**Affiliations:** 1 Infectious Diseases, Yale New Haven Hospital, New Haven, USA; 2 Infectious Diseases, University of California San Francisco Medical Center, San Francisco, USA; 3 Infectious Diseases, Vanderbilt University Medical Center, Nashville, USA

**Keywords:** gastrointestinal mucormycosis, invasive fungal disease, rhizopus oryzae, uncontrolled diabetes mellitus, unusual infection

## Abstract

Classically, mucormycosis is a known cause of rhino-orbital infection in immunocompromised patients. There is an increasing number of cases of mucormycosis infections involving locations outside of the rhino-orbital and pulmonary systems. While the factors that dictate these unusual locations of infection are not entirely known, physicians who encounter this scenario often resort to powerful antifungal medications and surgery to manage this disease process as there are no data to provide consensus on the best treatment approach. We share an interesting case of a patient who presented with diabetic ketoacidosis and was subsequently found to have gastrointestinal hemorrhage, with gastric ulcer biopsy revealing primary gastric mucormycosis.

## Introduction

Invasive mucormycosis is a life-threatening angio-invasive infection caused by fungi within the Mucoromycetes group. Classically, this entity is associated with severe sinopulmonary or rhino-orbital infections [1]. However, there are increasing reports of gastrointestinal (GI) mucormycosis, a rare manifestation of mucormycosis. These reported cases have involved locations that span virtually the entire GI tract and typically have several associated risk factors, including diabetes, prolonged neutropenia, and steroids [2-5]. Additionally, there have been reported cases of GI mucormycosis in immunocompetent patients without obvious risk factors as well as cases of post-COVID GI mucor [6-15]. While many of these patients receive a combination of antifungal and surgical management, the optimal management approach is unclear.

Here we present a case of GI mucormycosis in an adult male with diabetes. This case report highlights the salient clinical features of GI mucormycosis with an associated literature review.

## Case presentation

**Figure 1:**
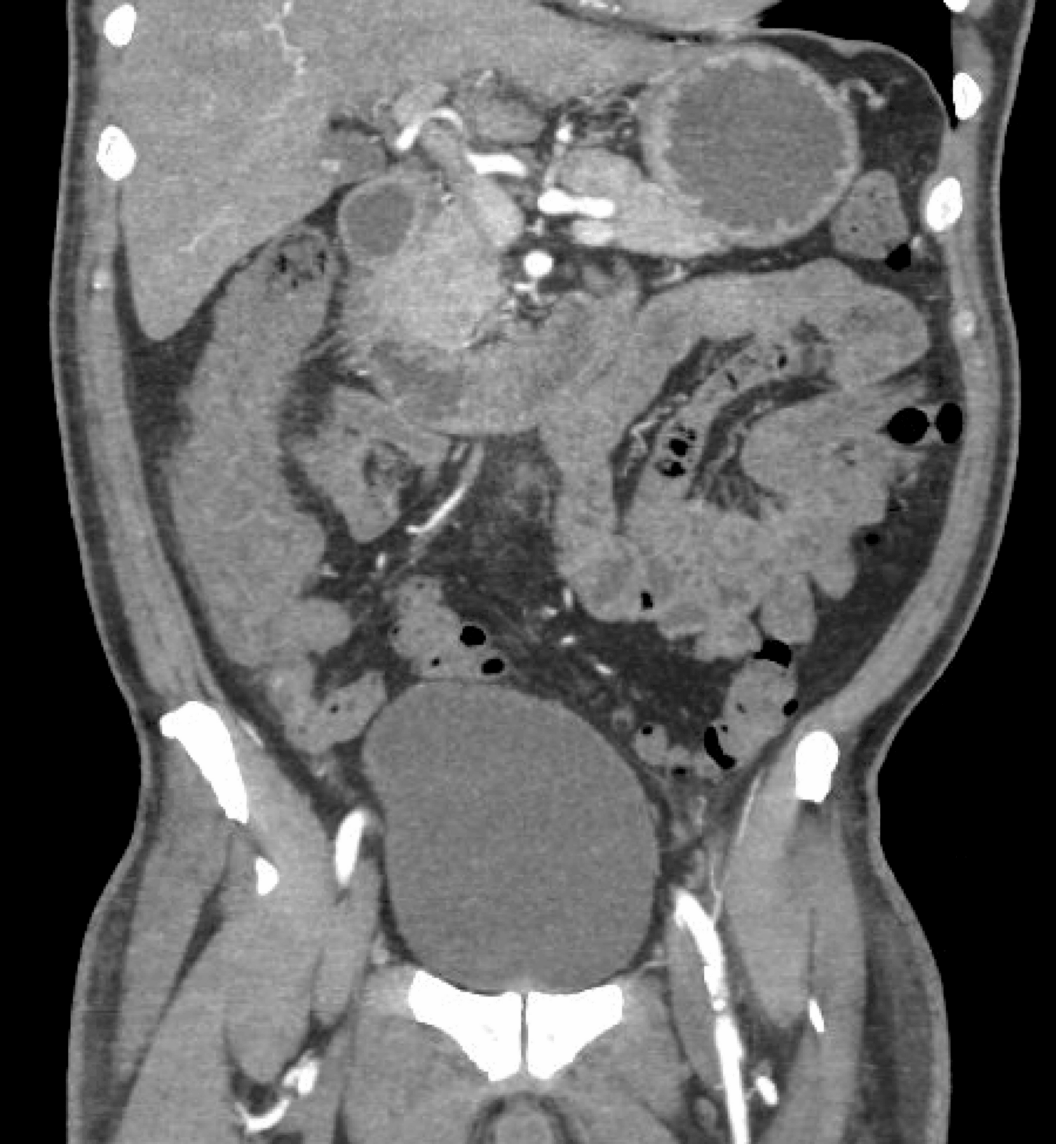
Coronal view of CT Abdomen & Pelvis showing colitis of ascending colon

A 52-year-old male with a prior history of uncontrolled type 2 diabetes (hemoglobin A1c 8.5) and hypertension initially presented for care due to acute bilateral vision loss and was found to have diabetic ketoacidosis (DKA). The patient reported that prior to symptom onset he had been drinking sodas and sugary drinks at home. He suffered a cardiac arrest approximately three hours after presenting with return of spontaneous circulation achieved after five minutes of resuscitation. He was then transferred to the ICU after being stabilized with transcutaneous pacing and initiation of norepinephrine. While in the MICU, he received vasopressor support, stress dose steroids, and continuous veno-venous hemofiltration (CVVH). On the second day of his admission, he developed a leukocytosis of 35.6 and developed diarrhea on day four. A C. difficile assay was negative and a CT abdomen/pelvis showed evidence of inflammation of the ascending colon (Figure 1). He underwent a cardiac catheterization on hospital day 4 which revealed severe left anterior descending (LAD) stenosis. CT chest revealed bilateral lower lobe and right middle lobe consolidations. He was started on vancomycin and piperacillin-tazobactam empirically, and subsequently received six days of piperacillin-tazobactam monotherapy before being transitioned to oxacillin after methicillin-susceptible Staphylococcus aureus (MSSA) grew in his sputum culture. 

**Figure 2:**
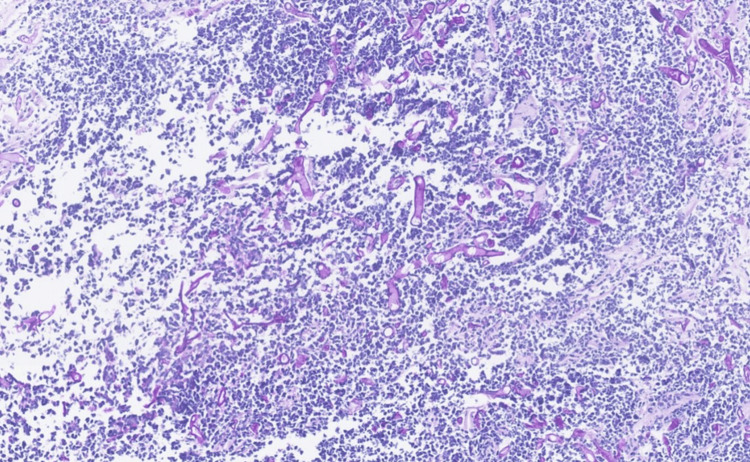
Periodic acid–Schiff (PAS) stain of gastric biopsy (400x)

His hemoglobin on admission was 11.5 and acutely dropped to 6.1 seven days into his hospitalization with the concurrent finding of melena. He required 2 units of blood and his heparin was held. An esophagogastroduodenoscopy (EGD) was ultimately performed and revealed multiple nonbleeding gastric and duodenal ulcers. Biopsies were obtained from multiple ulcers including one 20 mm ulcer on the gastric fundus that had a flat, pigmented spot, and heaped up and fibrotic borders concerning for malignancy. Overall, the gastroenterologist attributed findings of esophagitis and multiple ulcers to be most likely a result of recent ischemic injury. The pathology review of tissue biopsy noted necrotic debris likely from the ulcer bed that revealed numerous yeast forms with fungal hyphae most consistent with Zygomycetes (Figure 2). 

**Figure 3:**
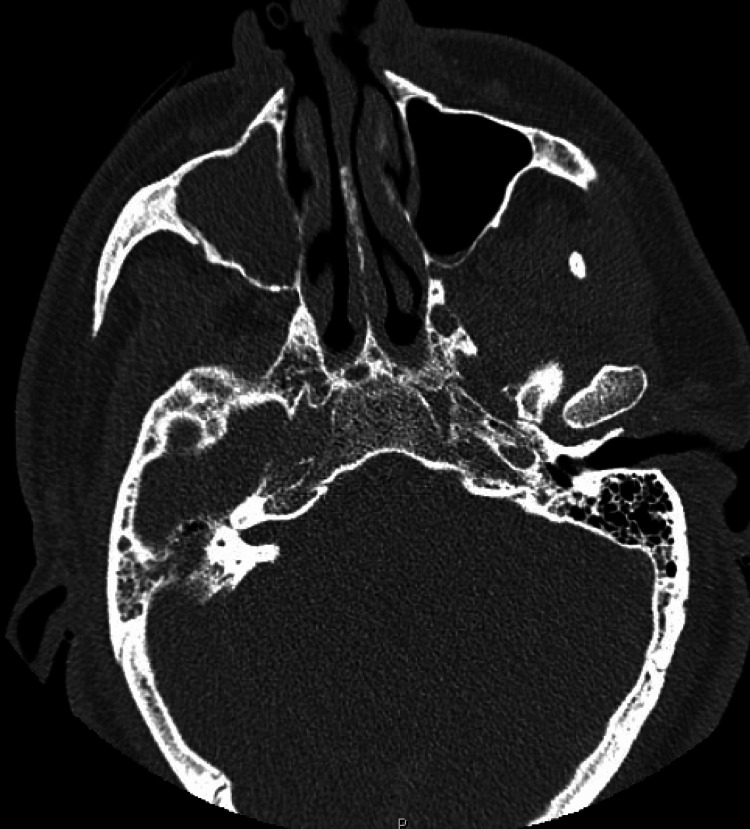
CT Sinus with contrast showing complete opacification of right maxillary air cells

At this point the infectious disease service was consulted. The patient was off vasopressor support, afebrile, and had been transferred out of the MICU. A CT head and neck was performed and showed pan-sinus disease with complete opacification of the right mastoid air cells and partial opacification of the right middle ear cavity (Figure 3). Flexible fiberoptic laryngoscopy revealed no evidence of necrotic lesions or invasive mucormycosis in the nasal mucosa or in the nasopharynx and no biopsy was performed. The decision was made to start induction therapy with liposomal amphotericin B. 

The patient continued to have episodes of symptomatic anemia and developed hematochezia again requiring transfusion and re-admission to the MICU for pressor support. Colonoscopy was deferred at that time due to concern for inducing iatrogenic perforation given his findings of colitis on CT scan. Due to this, the general surgery service was consulted and ultimately proceeded with exploratory surgery during which the patient’s colon and cecum were examined and noted to be without perforation. Two gastric wedge resections were performed, but these were unable to completely remove the ulcers that were noted from his previous EGD. The pathology report of the gastric wedge resections identified a single focus of submucosal granulomatous inflammation with fungal hyphae morphologically suggestive of mucor-like organisms. Ultimately, a PCR of the tissue pathology returned positive for Rhizopus oryzae. About one month into his stay, the patient underwent percutaneous coronary intervention (PCI) for his LAD stenosis. The patient's vision improved to near his baseline over this period of time as well. His renal function deteriorated after 27 days of liposomal amphotericin B and so the patient was changed to isavuconazonium and remained on this at discharge. Posaconazole was contraindicated due to the patient taking ticagrelor post-PCI. On follow-up it was discovered that the patient had been taking an oral probiotic prior to his hospitalization and was advised to discontinue this. He ultimately completed three months of total antifungal therapy. The patient achieved glycemic control with an A1c stable at 6.5, but he went on to experience additional hospitalizations for anemia and hematochezia with repeat EGD/colonoscopy six weeks later showing ulceration of his ascending colon without evidence of fungal invasion on biopsy. Another EGD/colonoscopy performed eight months from initial diagnosis showed healed gastric and colonic mucosa with biopsy revealing a benign well-differentiated neuroendocrine tumor in the colon, which was resected. The patient continues to be followed by Endocrinology and Gastroenterology.

## Discussion

Primary gastric mucormycosis is a rare manifestation of invasive Mucorales infection. One systematic review from 2019 indicated that gastric involvement accounted for 8% of cases and carries a poor prognosis with a 54% mortality rate [16]. The clinical presentations of GI mucormycosis can be non-specific, but cases frequently feature abdominal pain and fever with occasional melena, or even bowel perforation. These non-specific features may contribute to a delay in diagnosis [4]. Immunocompromised status, diabetes mellitus, and traumatic or iatrogenic injury have all been recognized as predisposing factors [16]. Notably, the level of free iron at an acidic pH in the serum of patients with DKA has been postulated to promote the growth of Rhizopus oryzae, in contrast to neutropenic patients who remain susceptible to mucorales infections due to lack of sufficient phagocytes [17].

The diagnosis of mucormycosis often depends on the identification of organisms in the tissue through histopathology. A high index of suspicion and early recognition of the disease are crucial, as timely diagnosis and treatment initiation have been shown to improve survival [18]. The application of PCR techniques can assist in diagnosis, but the lack of standardization and/or availability often limits utility [1].

Current management of mucormycosis usually involves a combination of surgical treatment and systemic antifungal therapy. Mucorales infections are characterized by angioinvasion of host tissue resulting in thrombosis and infarction [19]. As a result, surgical debridement is crucial for management due to the resultant necrotic tissue interfering with effective delivery of antifungal agents [5,17]. Liposomal amphotericin B monotherapy is currently recommended as first-line antifungal therapy for mucormycosis as it has an improved short-term nephrotoxicity profile compared with amphotericin deoxycholate [19]. The optimal duration of therapy for GI mucormycosis specifically is not known. Current available guidelines and expert opinion support treatment until resolution of symptoms and complete response on imaging, in addition to advising control or withdrawal of any immunosuppressing factors [1,20].

In this patient’s case, his uncontrolled diabetes predisposed him towards a Mucorales infection while the ischemic injury to his gastric mucosa likely disrupted the membrane integrity to a sufficient degree to allow fungal invasion. After a three-month course of antifungal therapy, the patient had a relatively good outcome considering the high mortality associated with this disease process. This was likely facilitated due to the Rhizopus invasion happening as an intercurrent process on top of the severe metabolic derangements and cardiovascular complications of the patient's hospitalization, for which the patient was already intensively being monitored. The identification of a colonic neuroendocrine tumor raises concern that this may have directly or indirectly contributed to the patient's hyperglycemia, although most of these malignancies are asymptomatic [21]. It is unclear if the probiotic supplement the patient was consuming prior to hospitalization had any role in the development of his disease. Only one other case has been reported by the CDC linking probiotic use in an infant to invasive Rhizopus oryzae infection of the GI tract [22]. The patient was advised to stop this probiotic out of an abundance of caution.

## Conclusions

GI mucormycosis is an unusual but often lethal infection that can occur in patients with controlled diabetes who are otherwise immunocompetent. Survival depends on timely detection and initiation of effective therapy as well as recruitment of surgical services. The case presented above likely had a good outcome due to his admission occurring prior to the onset of symptoms related to his GI infection. His extended hospitalization of over 40 days was due to multiple concurrent issues that needed stabilization prior to discharge. Given the increasing numbers of reported GI mucormycosis, it is imperative to consider this entity in appropriate hosts. 
